# The complete mitochondrial genome of *Sarcophila mongolica* (Diptera: Sarcophagidae)

**DOI:** 10.1080/23802359.2021.1884015

**Published:** 2021-03-11

**Authors:** Yaoqing Chen, Xiangyan Zhang, Yihong Qu, Lan He, Lipin Ren, Yadong Guo

**Affiliations:** a Department of Criminal Science and Technology, Hunan Police Academy, Hunan, Changsha, PR China; b Department of Forensic Science, School of Basic Medical Sciences, Central South University, Hunan, Changsha, PR China; c Department of Scientific Research Management Office, Hunan Police Academy, Hunan, Changsha, PR China

**Keywords:** Mitochondrial genome, *Sarcophila mongolica*, phylogenetic analysis

## Abstract

*Sarcophila mongolica* Chao & Zhang, 1988 (Diptera: Sarcophagidae) is considered to be of ecological and medical significance. In this study, we report the mitochondrial genome (mitogenome) of *S. mongolica*. This mitogenome was composed of 15,936 bp in length (GenBank accession no. MT845211), comprising 13 protein-coding genes (PCGs), two ribosomal RNAs (rRNAs), 22 transfer RNAs (tRNAs), and a non-coding control region. The arrangement of genes was identical to that of ancestral metazoan. Nucleotide composition revealed a strong A + T bias, accounting for 75.40% (A 38.2%, G 9.7%, C 14.9%, and T 37.2%). Phylogenetic analysis indicated that *S. mongolica* was obviously separated from the other flesh flies. This mitogenome provides important genetic data for further understanding of the evolutionary relationship within Sarcophagid flies.

Although the larvae of *Sarcophila* genus usually colonize on invertebrate carcasses, *Sarcophila mongolica* Chao & Zhang, 1988 (Diptera: Sarcophagidae) has so far been rarely reported except that the adult was found only in Inner Mongolia (Pape 1996; Xu and Zhao 1996). In this study, adult specimens were first trapped by pig liver in June 2019 from Ürümqi city (43°50′N, 87°37′ E), Xinjiang province, China. Whether they are of forensic importance or just accidental visitors remain unknown. All specimens were killed by freezing, and then identified by traditional morphological keys (Xu and Zhao 1996). These specimens were deposited at −80 °C in Guo’s lab (Hunan, Changsha, China) with a unique code (CSU19111977). Total DNA was extracted from thoracic muscle tissues of an adult specimen using QIANamp Micro DNA Kit (Qiangen Biotech Co., Ltd) according to the manufacture’s instruction. The genome sequencing of *S. mongolica* was performed on an Illumina HiSeq 2500 Platform, and then *de novo* assembly was carried out with MITObim version 1.9 and SOAPdenovo version 2.04 (https://github.com/chrishah/MITObim and http://soap.genomics.org.cn/soapdenovo.html) (Ren et al. 2020). Finally, the rough boundaries of all genes were initially identified by MITOS2 Web Server (http://mitos2.bioinf.uni-leipzig.de/index.py) (Ren et al. 2020).

In this study, the mitogenome of *S. mongolica* was 15,936 bp in length (GenBank accession no. MT845211), containing 13 protein-coding genes (PCGs), two ribosomal RNAs (rRNAs), 22 transfer RNAs (tRNAs), and a non-coding control region. The arrangement of genes was identical to that of ancestral metazoan (Cameron 2014). Nucleotide composition revealed a highly A + T bias, accounting for 75.40% (A 38.2%, G 9.7%, C 14.9%, and T 37.2%). Phylogenetic analysis of *S. mongolica* and other nine Sarcophagids species was constructed using maximum likelihood (ML) method based on the 13 PCGs, and *Calliphora vomitoria* (Diptera: Calliphoridae) was used as an outgroup (Figure 1). ML analysis was performed by IQ-TREE version 1.6.12 (Ren et al. 2020). The phylogenetic relationships indicated that the species of *S. mongolica* was clearly separated from the other flesh flies. This study provided important mitochondrial data for further studying on evolutionary relationships and species identification of flesh flies.

**Figure 1.**
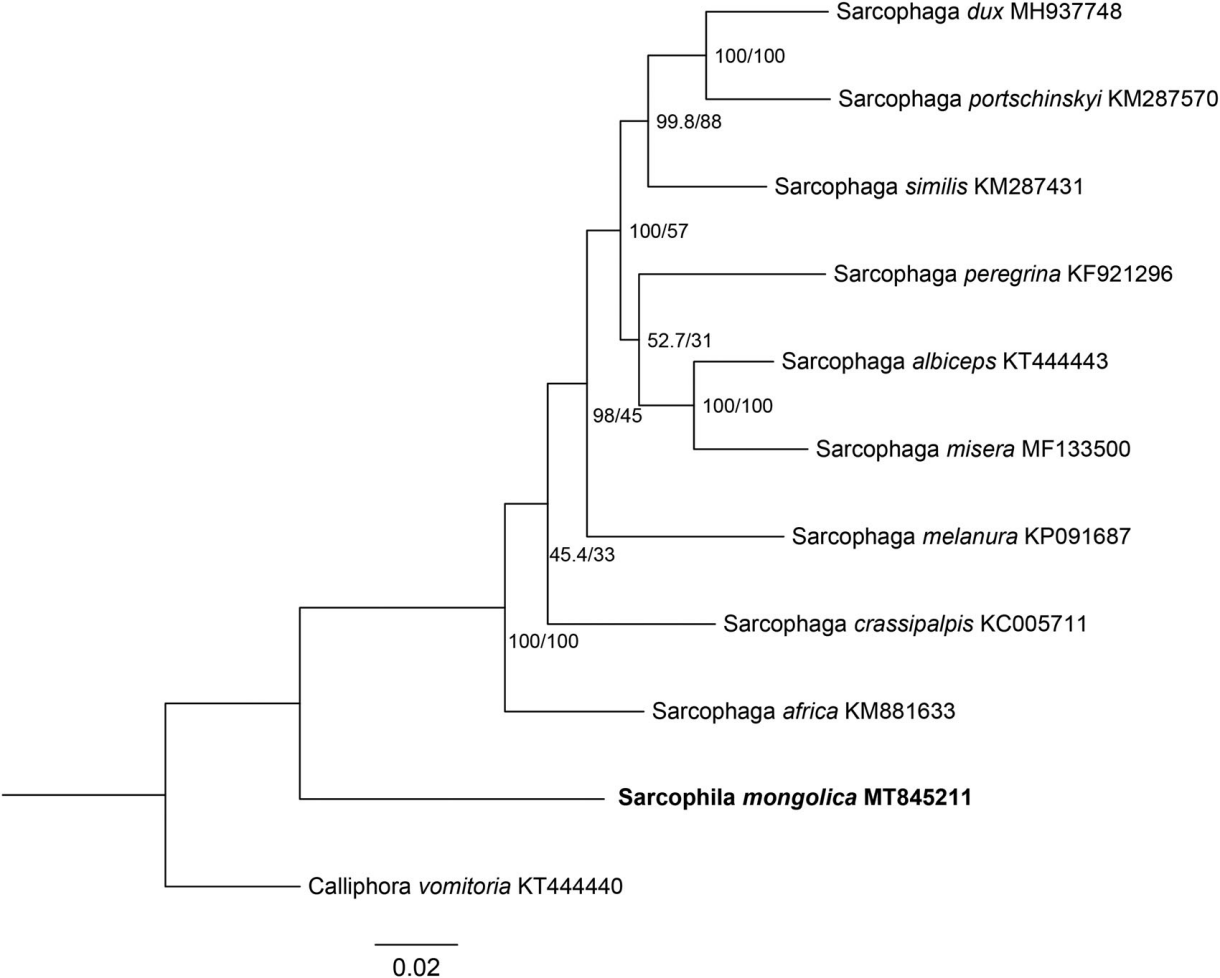
Phylogenetic trees of *S. mongolica* with nine sarcophagids species based on 13 PCGs by maximum likelihood (ML) method. *Calliphora vomitoria* was selected as an outgroup.

## Data Availability

Mitogenome data supporting this study are openly available in GenBank at: https://www.ncbi.nlm.nih.gov/nuccore/MT845211. Associated BioProject, SRA, and BioSample accession numbers are https://dataview.ncbi.nlm.nih.gov/object/PRJNA665806, https://www.ncbi.nlm.nih.gov/sra/SRR12719806, and SAMN16268934, respectively.
